# Validity of energy expenditure from combined accelerometry and heart rate during load carriage in adults with overweight to mild obesity

**DOI:** 10.1007/s00421-026-06230-9

**Published:** 2026-05-14

**Authors:** Joseph Dossou, Lina Majed, Nathan Townsend

**Affiliations:** College of Health and Life Sciences, Hamad Bin Khalifa University, Doha, Qatar

**Keywords:** Validity, Energy expenditure, ActiHeart 5, Weighted vest, Treadmill walking

## Abstract

The primary aim of this study was to examine the validity of energy expenditure (EE) estimated via branched chain modeling (BCM) during treadmill walking with a weighted vest. Thirteen sedentary adults with overweight/mild obesity [M = 11, F = 2; 29.0 (5.5) yr; maximal oxygen uptake (V̇O_2max_): 27.4 (2.0) mL·kg^−1^·min^−1^_;_ body mass index (BMI) = 29.6 (3.0)] were recruited. Participants completed a ramp incremental and steady-state walking (4.0, 4.5, 5.0, and 5.5 km·h^−1^) whilst wearing weighted vest (10% body mass; VEST) and unweighted control (CON). EE was estimated via indirect calorimetry (EE_IC_) and BCM (EE_BCM_), using a combined accelerometry/heart rate (HR) sensor (ActiHeart 5), and cardiorespiratory responses were determined from gas analysis. Limits of agreement (LoA) and concordance correlation coefficient (*CCC*) were used to assess validity of EE_BCM_. Repeated-measures ANOVA (condition_*_velocity) was used to examine physiological responses. Significant main effects of VEST included elevated oxygen consumption (V̇O_2_; ml.kg^−1^.min^−1^;*p* < 0.001), ventilation (*p* < 0.001), respiratory exchange ratio (RER; *p* < 0.001), HR (*p* < 0.001), and both EE_IC_ (*p* < 0.001) and EE_BCM_ (*p* < 0.001). There was a strong correlation between EE_IC_ and EE_BCM_ in both CON (*CCC* = 0.80) and VEST (*CCC* = 0.82). Mean bias (95% LoA) between EE_IC_ and EE_BCM_ was − 6.7 (-71.5,58.2) J·kg^−1^·min^−1^ (CON) and 3.6 (-50.3,57.6) J·kg^−1^·min^−1^ (VEST). Adults with overweight/mild obesity demonstrated an increase in EE during treadmill walking with a weighted vest. BCM was sensitive to this increase and also to treadmill velocity., However the agreement in estimated EE between indirect calorimetry and BCM was marginal for both CON and VEST conditions, and thus not interchangeable.

## Introduction

There is established evidence that links walking to healthy aging, including improved cardiovascular health, improved body composition and metabolic function, and decreased risk of falling (Bai et al. 2022). Furthermore, walking is the most common and accessible form of physical activity and constitutes the majority of movement-related energy expenditure (EE) at population scale (Siegel et al. 1995). This is especially true for individuals with overweight or mild obesity, since this population often experiences limited mobility due to musculoskeletal impairment and chronic or frequent pain e.g. low back pain which coalesce into a cycle of minimal or low intensity physical inactivity only (Arranz et al. 2014). For lifestyle modification programs aimed at weight reduction to be successful, accurate determination of EE is essential, not only for understanding how total daily EE (TDEE) is impacted, but also to assess how within day variations contribute to TDEE. For example, an increase in physical activity EE could be offset by longer periods of inactivity, referred to as “energy compensation” (Careau et al. 2021). Whilst doubly-labelled water remains the ‘gold standard’ for measurement of TDEE, this technique does not distinguish activity EE between daily living tasks and structured exercise of variable intensity (Argent et al. 2022; Westerterp 2018). Hence, the use of wearable sensor technology designed to estimate EE during walking or daily living activities in populations with overweight/mild obesity has received increasing attention in the literature (Strath et al. 2013). The most common methods to date have been either heart rate (HR) or accelerometry based, however in each case there are drawbacks which limit the validity of EE estimates (Chowdhury et al. 2017). One approach to address or overcome these limitations is to use ‘branched-chain accelerometry’ (BCM) which incorporates both HR and movement within the algorithm to estimate EE (Brage et al. 2004).

Wearable devices equipped with accelerometers estimate EE during ambulatory activities by tracking body movements in three axes (Van Hoye et al. 2014). To convert the raw measurement data, in units of m.s^− 2^ into units of EE, predictive equations have been developed. Some equations permit the calculation of EE as metabolic equivalents (METs) which is analogous to oxygen consumption (V̇O_2_), or as a rate of expenditure in joules or calories per unit of time (Lyden et al. 2011). However, the limitation of most accelerometers that rely solely on movement is that prediction equations may not provide accurate estimates of EE for a variety of activities. For example, movement only estimation of EE fails to account for changes due to factors such as changes in walking grade (including stairs), over variable terrain, and for load-bearing activities (Lyden et al. 2011). Energy expenditure estimation via HR on the other hand, is based on the assumed linear relationship with V̇O_2_ (Schantz et al. 2019). This method also contains drawbacks since research has demonstrated that at low exercise intensity, HR may not increase in direction proportion to EE, probably due to changes in stroke volume between lying, sitting, and standing (Amaro-Gahete et al. 2019). Furthermore, a dissociation between HR and EE can occur due to multiple factors, including physical activity type (cycling versus walking), locomotor muscle mass engaged, accumulated fatigue and exercise intensity which impact efficiency (Ndahimana and Kim 2017).

Combining HR and acceleration using a branched-chain model can improve the accuracy of EE estimation compared to either measure alone, as reported in several studies (Brage et al. 2007, 2015). The underlying principle is that integration of both measurements can attenuate the potential sources of error when estimating EE that either alone is susceptible to (Edwards et al. 2010). One such wearable device (ActiHeart 5, CamNtech), is a compact chest-worn monitor, which acts as a unipolar electrocardiograph, but also contains a tri-axial accelerometer which is thus fixed to the torso. This dual-sensor approach incorporates a branched-chain model has been employed in multiple laboratory-based studies involving different populations, settings, and activity types (Crouter et al. 2008; Klass et al. 2019), however few studies have specifically examined individuals with overweight/mild obesity. This population is often characterized by lower walking economy compared with normal weight individuals (Menéndez et al. 2019, 2020), which might introduce a systematic bias into estimates of EE from accelerometry alone. Furthermore, individuals with overweight/mild obesity also typically select slower walking speeds (Menéndez et al. 2019), which could limit the signal to noise ratio, in turn inflating the error of measurement for EE.

Given that individuals with overweight/mild obesity are more likely to experience limited mobility and maybe have difficulty to conduct exercise at high intensity, one easy and practical approach to elevate EE during physical activity, either structured or non-structured such as during daily living activities, is to add weight via load carriage. Furthermore, an intriguing hypothesis referred to as the “Gravitostat theory” has emerged in recent years based on evidence that increased chronic weight loading in rodents reduced fat mass and body mass in proportion to the weight added (Jansson et al. 2018). These findings were subsequently replicated in a proof-of-concept study (Ohlsson et al. 2020), and more recently a randomized clinical trial in humans with obesity (Bellman et al. 2025). The theory proposes a homeostatic mechanism acts to preserve gravitational load via regulation of fat mass independently of fat-derived leptin (Bellman et al. 2025; Jansson et al. 2018; Ohlsson et al. 2020). Also, a similar research design incorporating intentional weight loss with chronic weighted vest use, found significantly lower weight gain in the vest group following cessation of the weight loss program (DeLong et al. 2025). These findings are encouraging and support the use of a weighted vest for the purposes of weight management. However most studies which have examined EE during load carriage only investigated trained military personnel (Looney et al. 2022, 2024). Whilst these studies have generated a predictive equation referred to as the Load Carriage Decision Aid (LCDA), the purpose is specifically applied to an occupational/military setting and may not be appropriate to estimate EE during load carriage in sedentary individuals with overweight/mild obesity.

Whilst BCM using the ActiHeart device has been validated for total daily EE against the doubly labeled water technique (Silva et al. 2015), another study found poor agreement with activity EE within recreationally trained individuals (Santos et al. 2014). Accurate determination of activity EE is essential though, since this is the major component of total daily EE that can be modified via lifestyle intervention. During treadmill walking under well-controlled laboratory conditions, indirect calorimetry is considered the reference standard for estimation of EE, since it is sensitive to changes in substrate utilization, absolute exercise intensity, and can also be used to detect changes in relative intensity using manipulations that do not markedly alter the underlying movement pattern, such as exercise in hypoxia or load carriage (Argent et al. 2022). The objective of this study was to estimate EE via both indirect calorimetry (EE_IC_) and BCM using the ActiHeart device (EE_BCM_) during treadmill walking in a cohort of sedentary individuals with overweight/mildobesity. We also examined the effect of load carriage via the use of a weighted vest to assess whether EE_BCM_ was sensitive to an expected increase in HR without marked change to accelerometry. Lastly, we compared EE_IC_ and EE_BCM_ against the LCDA metabolic cost equation. We hypothesized that EE_BCM_ from the ActiHeart device would remain within acceptable limits of agreement compared to EE_IC_ during walking with, and without a weighted vest. A secondary hypothesis is that the LCDA will provide valid estimates of EE in sedentary adults with overweight/mild obesity as previously demonstrated for healthy military personnel (Looney et al. 2022, 2024).

## Methods

### Sample size calculation

This study was part of a larger project involving measurement of TDEE. Therefore, effect sizes were estimated for TDEE (estimated via actigraphy) from Chowdhury et al. (2017) which examined differences between various commercial accelerometry devices. Statistical power was set at 0.8 with an alpha of 0.05. Based on an effect size of 0.8 (strong) and a one-tailed distribution, the sample size was estimated at a minimum of 12 participants using G*Power software (version 3.1.9.6).

### Participants

Fifteen participants (13 males and 2 females) were initially recruited to participate in this study using mass emails, social networks and word of mouth. Two males voluntarily dropped out thus leaving a total of *N* = 13 for the completed data set. Participants were enrolled based on the following inclusion criteria: age (20–50 year), presenting a low physical activity levels according to standard guidelines (< 150 min·wk^− 1^ of moderate-intensity or < 75 min·wk^− 1^ of vigorous-intensity physical activity), categorized as overweight or obese according to body mass index (BMI = 25–35), but otherwise healthy. Exclusion criteria consisted of excessive obesity (BMI > 35), cigarette smokers, having a known history of any cardiovascular, metabolic, renal, pulmonary, musculoskeletal, or neural disorders, regular use prescription medication known to alter cardiorespiratory responses to exercise, or any current or recent musculoskeletal injury or surgery within the 3 months prior to the study. Before the study commenced participants were screened for exercise using the PAR-Q+ Physical Activity Readiness Questionnaire (Warburton et al. 2011), all procedures were explained, and written informed consent was obtained. The research was conducted according to the Declaration of Helsinki (World Medical Association 2025), and the experimental protocol was approved by the Hamad Bin Khalifa University Institutional Review Board (HBKU-IRB-2024-23).

### Experimental design

This study is a within-subjects (repeated-measures) experimental design with measurement validity. Participants visited a temperature (20.5 °C) and relative humidity (45%) controlled exercise physiology laboratory on two separate occasions separated by 3 to 7 days. The first visit was to become familiar with walking on a treadmill, typically 6–10 min depending on the participant (Meyer et al. 2019) and complete a ramp incremental walking test. The second visit involved walking on the treadmill at four different velocities (4.0, 4.5, 5.0, and 5.5 km·h^− 1^) for 4 min each, whilst wearing a weighted vest (VEST), or un-weighted control (CON). The weighted vest (Tunturi, Finland) was commercially available and fabricated from breathable nylon material with ergonomic shoulder straps and a total of eight front/back pouches containing sandbags. These were adjusted to generate the appropriate weight set at 10% of each participant’s own BW. This load was selected based on a combination of prior research (Ohlsson et al. 2020) and pilot testing to assess comfort level for longer duration use up to 10 h per day. The added weight was evenly balanced between the front and rear pouches. Rate of energy expenditure in J·kg^− 1^·min^− 1^ was estimated using branched-chain modeling of accelerometry and HR (EE_BCM_) and compared against indirect calorimetry as the reference standard (EE_IC_).

### Experimental protocol

#### Physical characteristics

On the first visit to the laboratory, BW and body composition (body fat percentage, fat mass and fat-free mass) were measured using a bioelectric impedance analyzer (MC 780 MA, Tanita, Japan), and height was measured using a wall-mounted stadiometer (Seca, Germany). In addition to pre-participation exercise screening, the long form of the International Physical Activity Questionnaire (Craig et al. 2003) was also completed to provide more accurate details of habitual physical activity levels. A validated non-exercise questionnaire to estimate maximal oxygen uptake (V̇O_2max_) was also completed (Nes et al. 2011). Participants then spent 10–15 min walking on the treadmill at self-selected speed up to 5 km.h^−1^ for familiarization ahead of the ramp incremental test.

### Treadmill exercise testing

All exercise tests were performed on a research-grade motorized treadmill (Katana XL, Lode, The Netherlands). Participants were instructed to refrain from caffeine and food for 3 h before testing, and refrain from any structured physical activity or alcohol consumption for 24 h prior.

*Ramp Incremental Test:* The ramp incremental walking test involved a 5 min baseline phase at 2.8 km·h^− 1^ immediately followed by a ramp phase in which the maximal velocity was limited to 5.8 km·h^− 1^. The ramp phase involved a simultaneous increase in both speed (0.10 km·h^− 1^) and gradient (variable) every 20 s such that the predicted V̇O_2max_ obtained from the questionnaire was attained within 8 to 12 min. This protocol was based on a previous study (Porszasz et al. 2003) whereby the increase in velocity is linear and the increase in gradient is curvilinear. This generates a linear increase in oxygen consumption until V̇O_2max_ is achieved which facilitates identification of exercise intensity domains via gas exchange thresholds. Participants were given verbal encouragement to produce a maximal effort, and the test was terminated upon volitional exhaustion.

*Steady-State Walking at Fixed Velocities:* Upon arrival at the laboratory participants completed an easy 5–8 min warm-up walking at 4.0 km·h^− 1^ to re-familiarize themselves with the treadmill. The test exercise bout comprised eight 4 min stages walking at four different constant velocities (4.0, 4.5, 5.0, and 5.5 km·h^− 1^) with, and without the weighted vest. The order of velocity was always incremental (i.e. starting at 4.0 km·h^− 1^) and the participants completed two stages at each velocity (with and without the vest) before increasing to the next velocity. The order of VEST and CON was counterbalanced, and there was a 60 s rest period between each stage to remove or replace the weighted vest.

### Data collection and analysis

*Gas exchange*: Respiratory gas exchange was measured breath-by-breath using a metabolic cart (COSMED Quark PFT, Italy). The gas analyzer and flow turbine calibrations were carried out before each test according to the manufacturer’s instructions using room air plus a physiological calibration gas (Buzwair, Qatar) a standard 3 L syringe (Hans Randolph, USA) respectively. The raw breath-by-breath data was initially filtered to remove outlier breaths due to swallowing, coughing etc. defined as greater than two standard deviations away from the 9-breath local mean and converted to 10 s bin averages thereafter. On the ramp incremental test, V̇O_2max_ was defined as the highest 30 s mean value in the event a plateau in O_2_ consumption occurred, or if peak HR was within 10 bpm of age predicted maximum *and* respiratory exchange ratio (RER) was > 1.10. All participants achieved these criteria. For the fixed velocity walking trials, all gas exchange data was averaged across the final 60 s of each stage. Nb: Steady-state oxygen consumption (V̇O_2_) and carbon dioxide production (V̇CO_2_) were confirmed by assessment of the slope of the breath-by-breath values versus time over the final 60 s, which was not significantly different to zero for any of the 4 min stages. Steady state V̇O_2_ and V̇CO_2_ were substituted into energy cost equations for moderate-intensity exercises (50–75%V̇O_2max_) as detailed in Jeukendrup and Wallis (2005) and converted from kcal·min^− 1^ to J·kg^− 1^·min^− 1^ thereafter for direct comparison to the ActiHeart data. Exercise ventilation (V̇E) is reported in L·min^−1^.

*Branched-chain modeling (BCM):* Energy expenditure was also estimated from a multi-sensor wearable device containing a tri-axial accelerometer and single-lead ECG (Actiheart 5, CamNtech, UK). Prior to exercise, a signal test was performed (10 min of wearing the device and light activity walking around the lab) to ensure high fidelity ECG waveform recording as recommended by the manufacturer (Kasper et al. 2023). The ActiHeart device samples the ECG waveform at 256 Hz and triaxial acceleration at 25 Hz, which are then imported after test completion into the manufacturer’s proprietary software (ActiHeart 5 version 5.1.10) for further analysis, which includes numerous model options available to estimate EE. For this study we chose the “group calibration/individual HR” model with branched mode options which combine a group mean component for movement derived from a previous validation study (Brage et al. 2007) with an individual HR calibration. For the latter, the corresponding HR for each participant’s EE determined from indirect calorimetry was entered into a calibration table within the software. We adopted this approach since this is the recommended option for the present population in a clinical scenario in which EE_IC_ is available. Analyzed data were exported thereafter as 15 s epochs and averaged over the same period as the gas exchange variables (i.e. last 120 s of each 4 min stage).

*Load Carriage Decision Aid (LCDA):* The supplemental digital content spreadsheet provided with Looney et al. (2024) was used to estimate energy expenditure (J·kg^− 1^·min^− 1^). This spreadsheet employs a model comprising body mass (kg), resting metabolic rate (W·kg^− 1^), vest load (% of body mass), walking speed (m·s^− 1^), gradient (%), and walking surface. The same variables were entered in the model for both conditions, with the exception of the added weight in VEST. The EE extracted from the LCDA is initially computed in units of Watts and then converted to J·kg^− 1^·min^− 1^.

*Heart Rate and Rating of Perceived Exertion:* HR was also measured using a conventional chest strap (Suunto, Finland) connected wirelessly via bluetooth to the metabolic cart data acquisition software. This data was averaged over the final 60 s of each stage during the steady-state walking session. Subjective rating of effort was determined via Borg’s Rating of Perceived Exertion (RPE) 0–10 scale (Borg 1982), immediately upon volitional exhaustion in the ramp incremental test and during the last 30 s of each stage in the steady-state walking session.

### Statistical analysis

All statistical analyses were performed using Jamovi version 2.3.26, an open-source R-Project interface with a significance level set at *p* < 0.05. The normality of all the data sets was verified using Shapiro-Wilks tests and visual inspection of Q-Q plots. Two-way repeated measures analysis of variance (ANOVA) was conducted to test for the interaction and main effects of condition (vest vs. control) and velocity (4.0, 4.5, 5.0, 5.5 km·h^− 1^) on physiological variables and the energy expenditure estimated using the ActiHeart 5 device and indirect calorimetry during the walking step test. The assumption of sphericity was verified, and Greenhouse-Geisser corrections were employed when a violation was reported. Where appropriate, post hoc comparisons were performed using Tukey’s HSD test. Partial eta-squared (η_p_^2^) is reported as the 2-way ANOVA main effect sizes interpreted as small ≈ 0.01, medium ≈ 0.06, and large ≈ 0.14 or higher (Cohen 1988). Mean bias and limits of agreement (LoA) between EE_IC_, EE_BCM_ and LCDA were assessed using Bland-Altman analysis, strength of association via repeated-measures correlation (for significance), and concordance correlation coefficient (for effect size). Repeated-measures correlation (*rm*) was chosen as it is well suited for validation studies involving repeated observations per participant, accounting for non-independence of data points and focusing on the consistency of the relationship within individuals over time (Bakdash and Marusich 2017). The concordance correlation coefficient (*CCC*) was used to assess the overall agreement between the two methods by evaluating both precision (proximity of data to the line of best fit) and accuracy (proximity of data to the line of identity). Strength of association for both the *CCC* and repeated-measures correlation are interpreted similarly to Pearson correlation as follows: negligible = 0.0–0.19.0.19, weak = 0.20–0.39, moderate = 0.40–0.59, strong = 0.60–0.79, and very strong = 0.80–1.0.80.0 (Altman 1991; Bakdash and Marusich 2017). All RPE data were non-normally distributed and therefore analyzed using the non-parametric Wilcoxon signed-rank test with a Bonferroni correction for multiple comparisons (i.e., CON vs. VEST at each velocity). All data is presented as mean and standard deviation. Statistical significance was accepted for *p* < 0.05.

## Results

Participants’ baseline physical and body composition characteristics are presented in Table 1.

### Physiological responses during steady-state treadmill walking

**Fig. 1:**
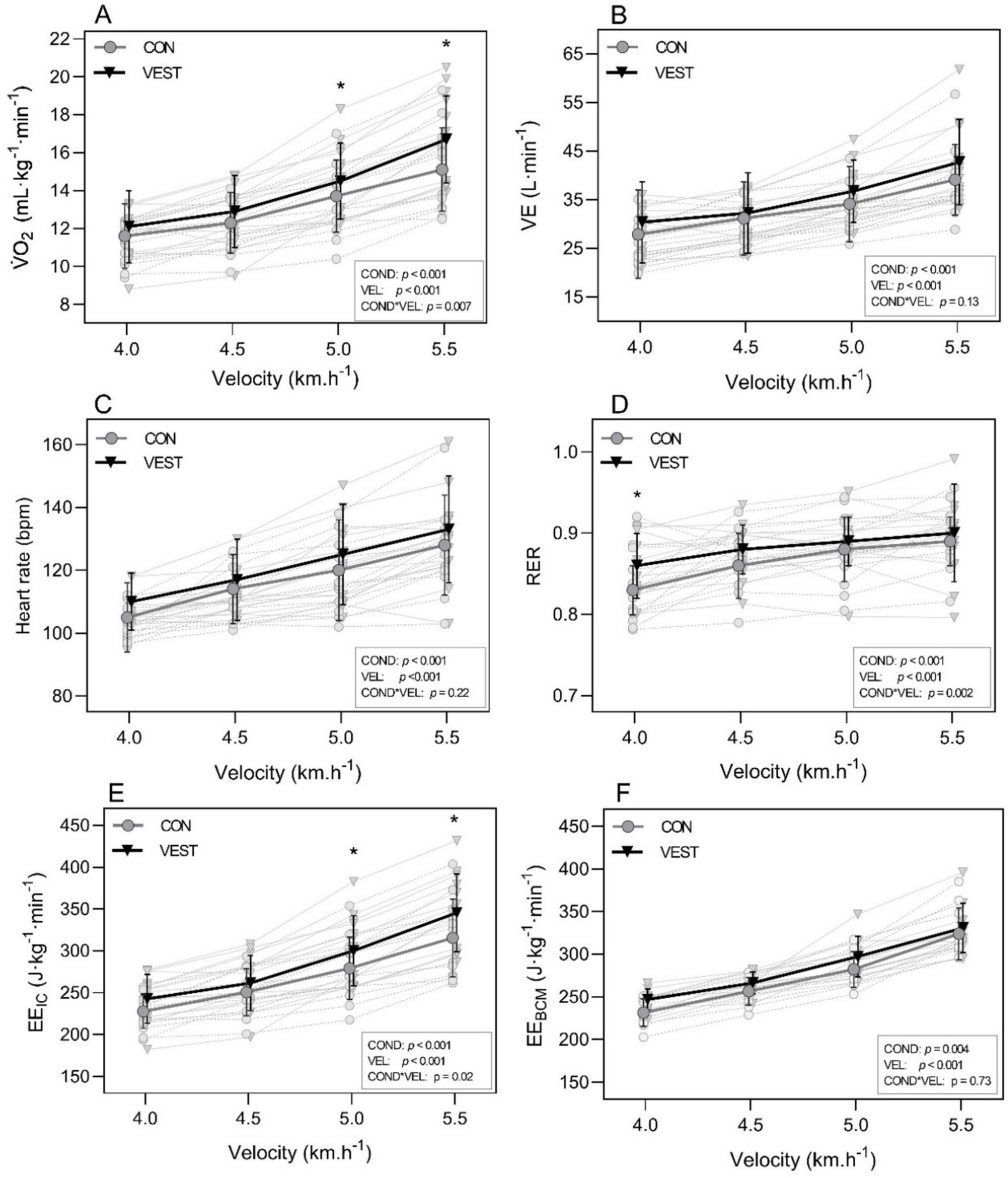
Physiological responses during constant velocity treadmill walking with (VEST) and without (CON) a weighted vest (10% body mass). *V̇O*_2_ oxygen consumption, *V̇E* pulmonary ventilation, *RER* respiratory exchange ratio, *EE*_IC_ gross energy expenditure via indirect calorimetry, *EE*_BCM_ gross energy expenditure via branched-chain model. Main effects *p* values for condition (COND), treadmill velocity (VEL) and the condition by velocity interaction (COND*VEL) are shown in each panel. Asterisks (*) denote post-hoc analysis significant differences (*p* < 0.05) at the velocity indicated

The effects of condition (CON, VEST) and velocity (4.0, 4.5, 5.0, 5.5 km·h^− 1^) on the physiological variables during treadmill walking are presented in Fig. 1. The range of relative intensity (%V̇O_2max_) was 43% − 56% for CON, and 45% − 62% for VEST. Two-way repeated measures ANOVA revealed a significant main effect of condition (*p* < 0.05) for all reported variables. As a percentage change in estimated marginal means (EMM) during VEST relative to CON, there was a 6.9% increase in V̇O_2_ (*p* < 0.001; η_p_^2^ = 0.77), an 8.6% increase in V̇E (*p* < 0.001; η_p_^2^ = 0.77), a 4.4% increase in HR (*p* < 0.001; η_p_^2^ = 0.66), a 2.0% increase in RER (*p* < 0.001; η_p_^2^ = 0.81), a 7.1% increase in EE_IC_ (*p* < 0.001; η_p_^2^ = 0.70), and a 4.0% increase in EE_BCM_ (*p* = 0.004; η_p_^2^ = 0.67). A condition by velocity interaction was also found for V̇O_2_ (*p* = 0.007; η_p_^2^ = 0.28), EE_IC_ (*p* = 0.008; η_p_^2^ = 0.35) and RER (*p* < 0.001; η_p_^2^ = 0.42). Post-hoc analysis revealed a 7.7% higher V̇O_2_ at 5.0 km·h^− 1^ (*p* = 0.046) and 9.7% higher at 5.5 km·h^− 1^ (*p* = 0.001) in VEST compared with CON, whereas no significant differences occurred at 4.0 km·h^− 1^ (*p* = 0.001) or 4.5 km·h^− 1^ (*p* = 0.001) A significantly higher RER was only observed at 4.0 km·h^− 1^ (*p* < 0.001) in VEST relative to CON. There was significantly higher RPE in VEST versus CON at 4.0 km·h^− 1^ (2.1 [0.7) vs. 1.4 [0.5]; *p* = 0.008), 4.5 km·h^− 1^ (2.9 [0.7) vs. 2.0 [0.8]; *p* = 0.048), 5.0 km·h^− 1^ (3.1 [0.7) vs. 2.5 [0.8]; *p* = 0.033), and 5.5 km·h^− 1^ (3.5 [0.7) vs. 2.7 [0.7]; *p* = 0.011), compared with CON.

### Energy expenditure: accelerometry versus indirect calorimetry

**Fig. 2:**
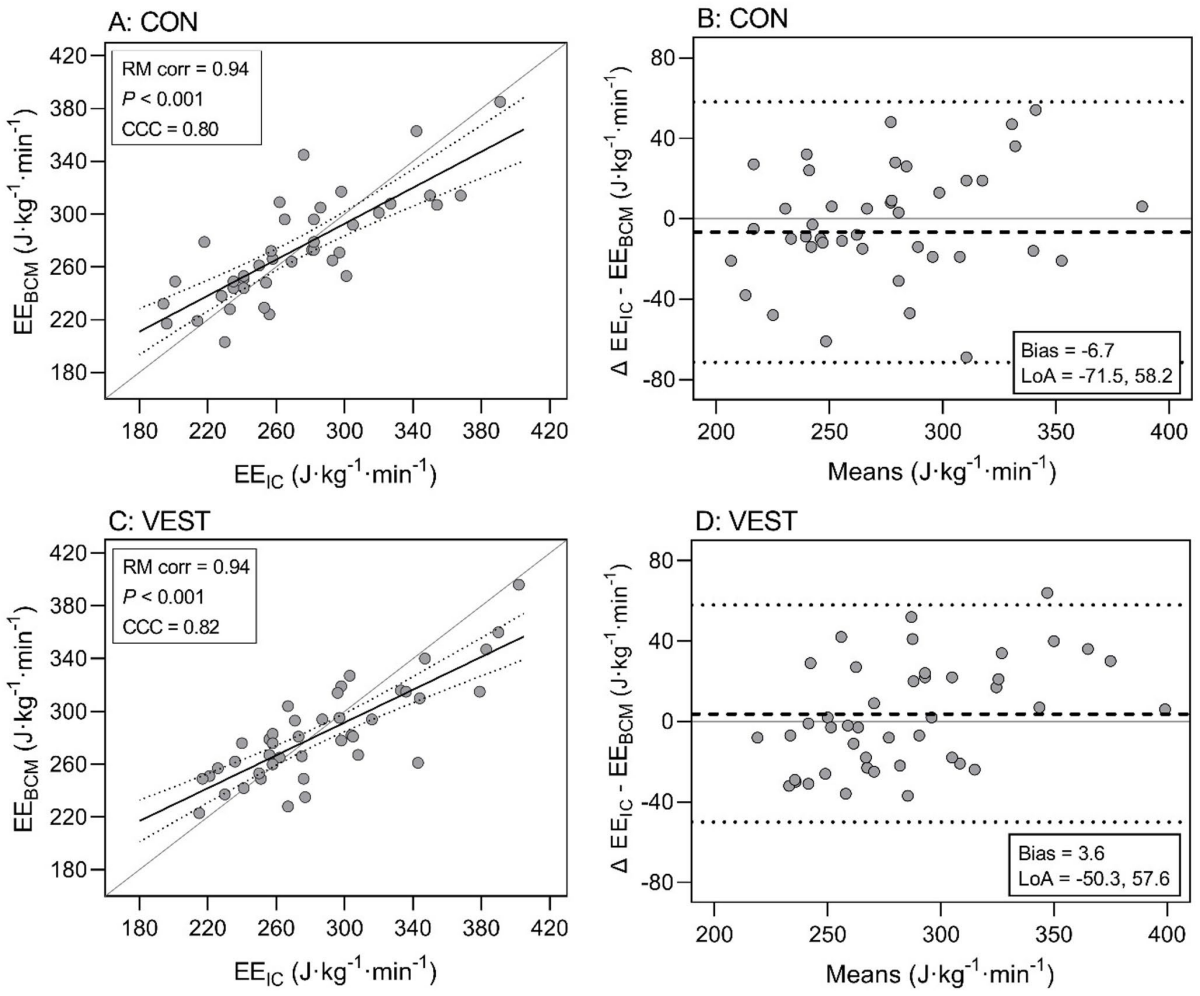
Line of identity and Bland-Altman plots showing agreement between gross energy expenditure (EE in units of J·kg^− 1^·min^− 1^) estimated via indirect calorimetry (EE_IC_) and accelerometry branched chain model (EE_BCM_), during both unweighted (CON; **A** and **B**) and weighted (VEST; **C** and **D**) treadmill walking. **A** and **C**, the light grey line denotes the line of identity. **B** and **D**, the thick black dashed line represents mean bias, and the dotted lines show upper and lower limits of agreement (LoA). Nb: *RM* *corr* repeated measures correlation coefficient. *CCC *concordance correlation coefficient

A 2-way ANOVA revealed no significant difference for the main effect of method (EE_IC_ vs. EE_BCM_) during the treadmill test (*p* = 0.96; η_p_^2^ < 0.001). Line of identity and Bland-Altman plots during steady-state treadmill walking in CON and VEST conditions are presented in Fig. 2. Repeated measures correlation revealed a strong positive relationship between EE_IC_ and EE_BCM_ for both CON (*rm* = 0.94; *p* < 0.001) and VEST (*rm* = 0.94; *p* < 0.001). Also, the *CCC* is classified as “very strong” (*CCC* > 0.8) for both conditions. Mean bias was close to 0 J·kg^− 1^·min^− 1^ in both conditions, however, the LoA was large, with a percentage error of 48% and 40% for CON and VEST respectively.

### Comparison with the Load Carriage Decision Aid (LCDA)

**Fig. 3:**
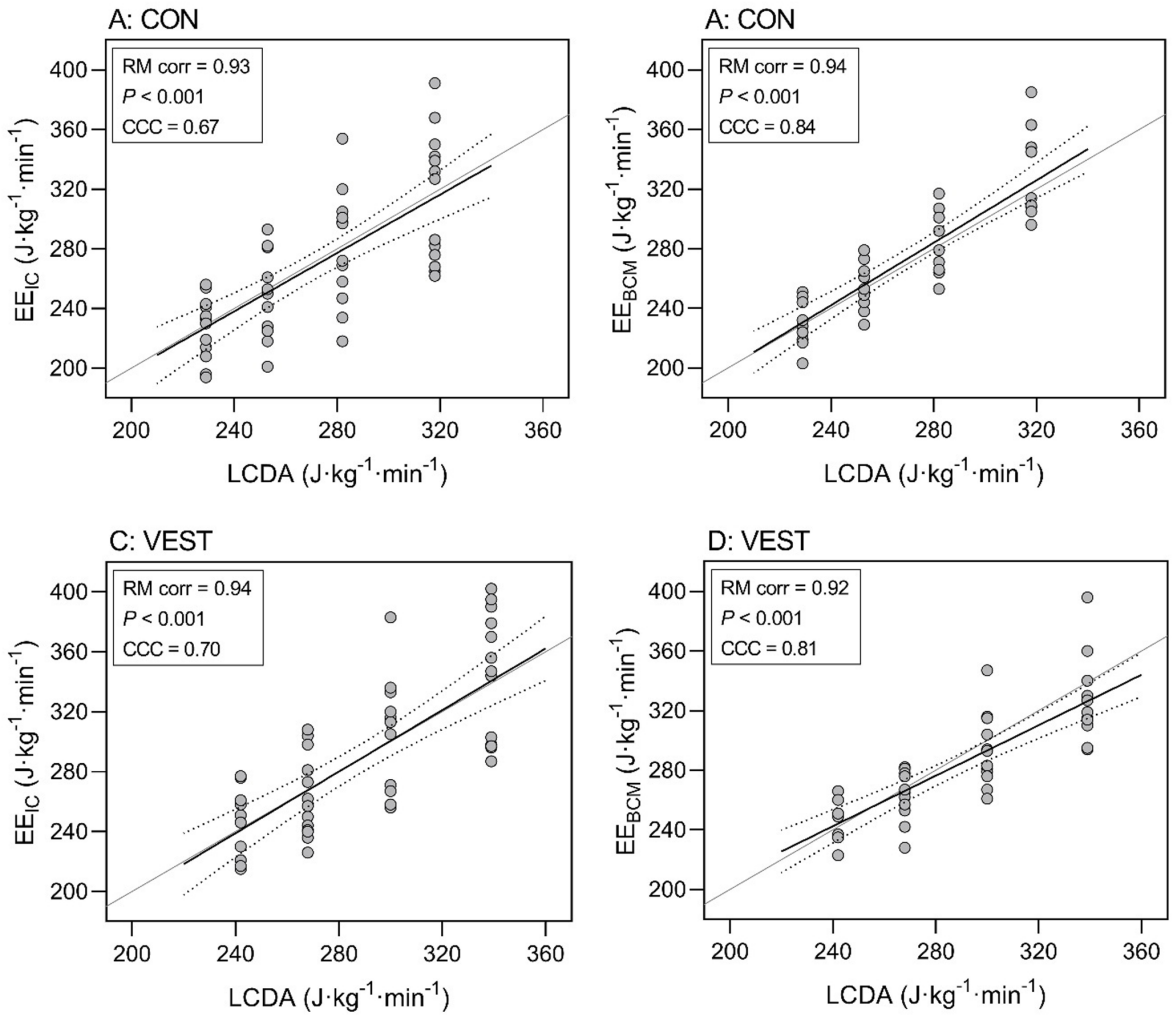
Line of identity plots showing the relationship between gross energy expenditure (EE in units of J·kg^− 1^·min^− 1^) estimated via indirect calorimetry (EE_IC_), accelerometry branched chain model (EE_BCM_), and the Load Carriage Decision Aid (LCDA) metabolic cost equation during both unweighted (CON; **A** and **B**) and weighted (VEST; **C** and **D**) treadmill walking. Nb: In each panel, the x and y axis units are not identical, and therefore the light grey line of identity is offset from the 45° diagonal. Note also that since the LCDA is mainly a function of BW and treadmill speed, and units for EE are normalized to BW, the x-axis resolves to discrete values for each treadmill stage. *RM corr* repeated measures correlation coefficient, *CCC* concordance correlation coefficient

A 2-way ANOVA revealed no significant main effect for EE_IC_ vs. LCDA (*p* = 0.80; η_p_^2^ = 0.008) or EE_BCM_ vs. LCDA (*p* = 0.59; η_p_^2^ = 0.03) during the treadmill test. The line of identity plots for estimates of EE from each method and condition is presented in Fig. 3. Repeated measures correlation demonstrated a strong positive relationship (*rm* > 0.9) between both EE_IC_ and EE_BCM_ versus the LCDA estimated value. The *CCC* for EE_IC_ versus LCDA is classified as “strong” for CON (*CCC* = 0.67) and VEST (*CCC* = 0.70), and “very strong” for EE_BCM_ versus LCDA for CON (*CCC* = 0.84) and VEST (*CCC* = 0.81).

## Discussion

This study assessed physiological responses and EE during treadmill walking with, and without a weighted vest (10% BW) in a cohort of sedentary adults with overweight/mild obesity. We compared EE estimated via three methods: (1) indirect calorimetry, (2) an accelerometry + HR branched chain model, and (3) the LCDA prediction equation. Wearing the weighted vest induced a measurable increase in V̇O_2_ and EE, with greater increases at higher walking speeds. There were strong positive correlations and low mean bias between all methods to estimate EE across a range of velocities (4.0–5.5 km·h^−1^) which are thus near the typical preferred walking speed for adults with mild obesity (Fernández Menéndez et al. 2019). Consequently, use of a research-grade field-based accelerometer (ActiHeart 5) showed sufficient precision to detect differences in activity EE in the VEST condition. However, compared to the criterion method of indirect calorimetry, the estimated EE from both branched-chain modeling and the LCDA demonstrated relatively wide limits of agreement. These results imply that BCM of accelerometry plus HR can be used to reflect differences in EE due to relatively minor changes in walking velocity and load carriage, but that BCM should not be used interchangeably with indirect calorimetry or the LCDA at the individual level.

Few studies have examined changes in EE during weighted vest walking in untrained overweight participants, with a majority of the literature focused on military personnel (Straight et al. 2025; Vickery-Howe et al. 2025), occupational settings (Lyons et al. 2005), and in exercise training (Martínez-Noguera et al. 2024; Purdom et al. 2021). In our cohort of overweight sedentary adults, we observed a ≈ 7% increase in the rate of EE whilst wearing a weighted vest, ranging from a 6.1% increase at 4.0 km·h^− 1^ up to 9.7% at 5.5 km·h^− 1^. In a comparable study by McCormick et al. (2015), untrained young adult females wore a weighted vest (10% and 15% BM) during treadmill walking at 4.03 km·h^− 1^ across several gradients (0, 5, 10, and 15%). These investigators observed non-significant changes in V̇O_2_ with 10% added BW at 0% gradient, which is similar to the present study, and a trend towards a greater effect with higher gradients and added weight (15% versus 10% BW). Given that in our study we did not manipulate the gradient, but only increased the treadmill velocity, the combined results suggest the interaction effect between and added weight and exercise intensity on increasing EE is not specific to changes in gradient or velocity alone. The average relative intensity in our study ranged from ≈ 43% to 62% of V̇O_2max_. These intensities are lower than another study, which examined the effect of a weighted vest (5% and 10% BW) during treadmill running at several velocities equivalent to 60 to 80% of V̇O_2max_ (Purdom et al. 2021). These investigators reported a significant increase in EE with 10% BW added, but not 5%, and there was no interaction effect between the weighted vest and intensity. During walking with graded increases in load carriage, there appears to be a linear effect on the energy cost of transport (Huang & Kuo 2014), however, a nonlinear increase occurs as walking speed moves beyond the most economical velocity, which occurs around 4–4.5 km.h^− 1^ for net EE (Abe at al. 2015). Hence, there may be an interaction between load carriage and declining biomechanical efficiency, which leads to a non-linear effect of on EE with increasing velocity, particularly as the walk-to-run transition is approached. Further research is required to confirm these results and test this hypothesis though.

Accurate assessment of total daily EE is essential for understanding energy balance, metabolic health, and obesity-related risk in sedentary adults with overweight. Whilst the gold standard for this measure is considered to be doubly-labelled water technique (Westerterp 2018), this method is not practical for routine assessment of EE in the general population, and is insensitive to changes specifically in activity EE alone (Santos et al. 2014). Whilst indirect calorimetry can accurately determine acute changes in activity EE, this method is also impractical for routine use during daily living. Thus, wearable sensors, incorporating HR and accelerometry have emerged as viable alternatives to estimate EE outside of the laboratory environment (Chowdhury et al. 2017). The BCM algorithm used in the present study involves weighting the relative contributions of accelerometry and HR to determine EE. More specifically, when both activity and HR values are low, the BCM algorithm proportionally favors EE estimated form accelerometry, whereas for higher exercise intensity the HR algorithm predominates (Crouter et al. 2008). In a validation study on the assessment of EE via ActiHeart 5, a strong correlation (*r* = 0.92) with indirect calorimetry during running at 60–85%V̇O_2max_ was reported (Klass et al. 2019). These investigators noted ≈ 3–20% lower EE values though for the ActiHeart compared to indirect calorimetry. In another study, also using indirect calorimetry as the reference standard, EE estimated from the ActiHeart device showed a strong correlation (*r* = 0.88) when averaged across a range of velocities from 3.6 up to 9.6 km.h^−1^ which thus includes running (Barreira et al. 2009). The use of repeated-measures correlation is preferable though to averaging multiple data points (i.e. velocities in this instance) since the latter distorts the true measurement variance. Using this statistical method, we observed an even higher correlation coefficient between EE_IC_ and EE_BCM_ (*rm* = 0.94 for both CON and VEST; Fig. 2). Conversely, one limitation of repeated-measures correlation statistical tool is that it is not sensitive to mean bias between two measurement tools. The *CCC* does however take this account and consequently the strength of association was slightly lower (*CCC* = 0.80 for CON and 0.82 for VEST) yet remained strong. As mentioned, the relative intensity range in the present study was ≈ 43% to 62% of V̇O_2max_, resulting from treadmill velocities which remained close (≈ ± 1.5 km·h^−1^) to the preferred walking speed for individuals with mild obesity (Fernández Menéndez et al. 2019). Hence, our results broadly confirm those of other reports on different participant samples and extend also to overweight/mild obesity.

To fully comprehend methodological validity, Bland-Altman “limit of agreement” analysis is recommended (Bland and Altman 1986). Using this analysis, we found a trivial mean bias between EE_IC_ and EE_BCM_, which was slightly negative for CON and positive for VEST (Fig. 2). The 95% LoA between EE_IC_ and EE_BCM_ was approximately ± 50–60 J·kg^− 1^·min^− 1^ which equates to ± 270–324 kJ per hour for a 90 kg individual (i.e. similar to the mean BW for the present cohort). These limits would be considered wide from a clinical perspective since an error of ≈ 300 kJ for a one-hour walk represents a significant proportion of total daily EE in minimally active individuals. A limitation of the present study design though, is the relatively short duration (60 s) used to average V̇O_2_ and V̇CO_2_, which can be impacted by short-term modulation of pulmonary V̇E during exercise (Wood et al. 2008). However, the low mean bias across multiple exercise intensities suggests “regression to the mean” could have occurred. Therefore, we recommend that future validation studies should include measurements taken across longer durations typical for structured exercise (e.g. multiple bouts of 5–10 min) or across an entire day as per normal living activities (e.g. using portable gas analysis devices). Our findings are broadly consistent with previous work showing comparable limits of agreement in the adult population during unloaded walking trials (Chowdhury et al. 2017; Edwards et al. 2010; Klass et al. 2019). These broad LoAs likely reflect individual differences in physiology which impact the activity EE, but are not accounted for by either accelerometery or HR alone. For example, substrate utilization, stroke volume, haemoglobin concentration, and biomechanical and metabolic efficiency (Brage et al. 2004). Despite the relatively wide LoA between EE_IC_ and EE_BCM_, overall the results from the present investigation indicate the ActiHeart 5 device demonstrates practical utility for estimating activity EE, within individuals with overweight/mild obesity. Moreover, the sensitivity to the change in EE due to the weighted VEST (i.e. a condition which mainly alters HR) aligns with previous research which indicates that BCM is superior to accelerometry (or HR) based methods alone (Brage et al. 2004, 2007). The practical implication here is that wearable sensors designed to estimate EE should incorporate both accelerometry and HR. For example, some low cost, easy to use consumer grade HR monitors include a triaxial accelerometer (e.g. Polar H10), however there is no application presently available designed to extract both acceleration and HR data.

An alternative non-invasive method to estimate EE is via the use of a walking metabolic cost equations. Numerous prediction models have been developed for the purpose of estimating EE during locomotion, however there are few with a term which encompasses load carriage. The Load Carriage Decision Aid (LCDA), developed by the U.S. Army Research Institute of Environmental Medicine (USARIEM), was designed to estimate total EE in situations where the use of indirect calorimetry or activity trackers is not routinely available (Looney et al. 2022, 2024). Although the energetic requirements of the population we studied in our trial may vary from a military population used in deriving the LCDA coefficient, this model appeared to be valid for estimation of EE during loaded and unloaded walking in our sedentary overweight/mild obese cohort. By design, the LCDA model predicts higher EE with additional load carriage, and we found no significant differences for EE between LCDA and either EE_IC_ or EE_BCM_. We also observed a strong repeated-measures correlation (which is sensitive to the change in EE due to increasing treadmill velocity) between EE estimated from LCDA and both EE_IC_ and EE_BCM_ (Fig. 3), in both CON and VEST conditions. Finally, the *CCC* for the LCDA vs. EE_IC_ was classified as ‘moderate’ (*CCC* = 0.67 for CON and 0.70 for VEST). Interestingly, despite the slightly lower *CCC* in this instance (relative to EE_IC_ versus EE_BCM_ shown in Fig. 2), the regression line was very close to the line of identity. This indicates the ActiHeart device displayed lower variance compared to the indirect calorimetry reference than the LCDA, which is not surprising though since it involves direct measurement of both the individual movement and HR. In contrast, the slightly higher *CCC* values between EE_BCM_ and LCDA (*CCC* = 0.84 for CON and 0.81 for VEST), likely reflect the common use of predictive equations in each case. The practical implication of these results is that whilst the LCDA is inexpensive and may be useful for prognostic evaluation of expected energy requirements “in the field”, the use of a wearable sensor incorporating accelerometry and HR is superior for estimating what was actually performed, in particular EE fluctuations throughout the day due to time spent sitting, performing daily activities, and exercising.

### Methodological considerations

In the present study we estimated EE using the manufacturer’s proprietary analysis software (ActiHeart 5 version 5.1.10, CamNtech, UK). We applied an individual calibration of HR versus activity EE in combination with a previously derived equation which computes the accelerometry to EE relationship (Brage et al. 2004). Initially the BCM was first developed and validated against EE using a 24 m^3^ direct calorimeter chamber (Brage et al. 2004), and thereafter to enhance the practical utility of BCM, a number of different calibration methods were developed (Brage et al. 2007). The investigators reported the lowest standard error of the estimate for BCM_EE_ when derived from the individually measured HR versus EE relationship. Thus, in the present study we wished to adopt the “best case scenario” which represents the practical application to laboratory based research studies and the clinical environment in which a metabolic cart is available. However, it was also reported by Brage et al. (2007), that only marginally lower model precision (R^2^ = 0.977 vs. 0.947) could be achieved using group mean equations derived previously (Brage et al. 2004). The authors suggested this alternative calibration procedure could thus be adopted for large scale epidemiological studies in which individual calibration may not be feasible. The similarity in model precision between individual and group level calibration suggests results from the present study can be approximated to “real world” settings in which a metabolic cart may not be available.

## Conclusion

This is the first study which examined EE determined via combined accelerometry and HR using an experimental design in which EE was altered independently of the movement pattern. We achieved this by using load carriage (a commercially available weighted vest). The branched-chain model was sensitive to the change in EE due to the added weight and showed a strong correlation with EE derived from indirect calorimetry in both the CON and VEST conditions. Whilst the LoA were broad, the mean bias was trivial which suggests that within-subject variance over short timescales (e.g. as measured in the present study) might decrease over longer duration used to estimate total daily EE, although this requires further research. We also found consistent increases in absolute EE, V̇O_2_, HR, and V̇E with load carriage even though the added weight was relatively modest (10% of BW) for comfortability. Thus, relative intensity was increased in all participants, and this effect was more pronounced at faster walking speeds. In summary, the ActiHeart device is a valid tool to estimate activity EE during walking in both loaded and unloaded scenarios. The use of added weight significantly increased/elevated activity EE and thus represents a simple intervention to increase relative exercise intensity in adults with overweight and mild obesity who may otherwise experience limited mobility.

**Table 1:** Participant physical characteristics

Variables	Male	Female	Pooled
Number	11	2	13
Age (Years)	29.5 (5.8)	26.0 (1.4)	29.0 (5.5)
Body Mass (kg)	93.5 (9.3)	85.9 (0.3)	92.3 (8.9)
Height (cm)	176.1 (10.6)	167.0 (2.8)	175.0 (10.3)
BMI (kg·m^− 2^)	29.4 (3.2)	30.8 (1.1)	29.6 (3.0)
Body Fat (%)	26.3 (4.7)	36.7 (5.7)	27.9 (6.0)
Fat-Free Mass (kg)	63.8 (12.7)	38.2 (14.1)	59.9 (15.6)
Fat Mass (kg)	24.9 (6.3)	31.6 (4.7)	26.0 (6.4)
V̇O_2max_ (L·min^−1^)	2.50 (0.20)	2.39 (0.02)	2.45 (0.17)
V̇O_2max_ (mL·kg^−1^·min^−1^)	27.3 (2.50)	28.4 (2.34)	27.4 (2.03)

## Data Availability

The datasets generated and analyzed during the current study are not publicly available due to IRB privacy and confidentiality requirements. The datasets can be obtained from the corresponding author upon request.
